# Sex differences in pediatric caudal epidural anesthesia under sedation without primary airway instrumentation

**DOI:** 10.1371/journal.pone.0288431

**Published:** 2023-07-13

**Authors:** Philipp Opfermann, Werner Schmid, Mina Obradovic, Felix Kraft, Markus Zadrazil, Daniela Marhofer, Peter Marhofer

**Affiliations:** Department of Anesthesia, General Intensive Care Medicine and Pain Medicine, Medical University of Vienna,Vienna, Austria; University Hospital Knappschaftskrankenhaus Bochum, GERMANY

## Abstract

**Study objective:**

To identify sex differences associated with caudal epidurals, the most commonly used technique of pediatric regional anesthesia, based on individually validated data of ultrasound-guided blocks performed between 04/2014 and 12/2020.

**Methods:**

Prospectively collected and individually validated data of a cohort of children aged between 0–15 years was analyzed in a retrospective observational study. We included pediatric surgeries involving a primary plan of caudal epidural anesthesia under sedation (without airway instrumentation) and a contingency plan of general anesthesia. Sex-specific rates were analyzed for overall failure of the primary anesthesia plan, for residual pain, for block-related technical complications and for critical respiratory events. We used Fisher´s exact tests and multivariable logistic regressions were used to evaluate sex-specific associations.

**Results:**

Data from 487 girls and 2060 boys ≤15 years old (ASA status 1 to 4) were analyzed. The primary-anesthesia-plan failure rate was 5.5% (95%CI 3.8%-7.8%) (N = 27/487) among girls and 4.7% (95%CI 3.9%-5.7%) (N = 97/2060) among boys (p = 0.41). Residual pain was the main cause of failure, with rates of 4.5% (95%CI 2.9–6.6%) (N = 22/487) among girls and 3.0% (95%CI 2.3–3.8%) (N = 61/2060) among boys (p = 0.089). Block-related technical complications were seen at rates of 0.8% (95%CI 0.3%-1.9%) (N = 4/487) among girls *vs* 2.5% (95%CI 0.5–2.7%) (N = 51/2060) among boys and, hence, significantly more often among male patients (p = 0.023). Male sex was significantly associated with higher odds (adjusted OR: 3.18; 95% CI: 1.12–9; p = 0.029) for such technical complications regardless of age, ASA status, gestational week at birth or puncture attempts. Critical respiratory events occurred at a 1.7% (95%CI 1.2%-2.3%) rate (N = 35/2060) twice as high among boys as 0.8% (95%CI 0.3%-1.9%) (N = 4/487) among girls (p = 0.21).

**Conclusions:**

While the the primary-anesthesia-plan failure rate was equal for girls and boys, technical complications and respiratory events are more likely to occur in boys.

## Introduction

Personalised medicine, with a strong focus on individual patient needs, is going to become a widely accepted practice in the foreseeable future. Part and parcel of this development is a solid understanding of sex differences, and there is considerable advocacy in the scientific community to promote such knowledge [1]. In the field of anesthesia, however, the role of sex differences has given to less attention in the past [2].

An impact of patient sex on the effect and dosage of anesthetic agents may be present because of pharmacokinetic differences associated with different body compositions, but also as a consequence of metabolic differences in elimination pathways [3]. For example, females are less sensitive to propofol presumably manly due to pharmacodynamic mechanisms, but more sensitive to opioids most likely to pharmakodynamic effects [4].

Even though numerous factors contribute to individual patients’ levels of sensitivity or risk profiles in their response to anesthesia, neglect to systematically include and analyse female subjects in anesthesia research may, in the past, have masked the existence of relevant sex differences [3]. Geller and coworkers conducted a study to evaluate the compliance with inclusion and assessment of women and minorities in National Institutes of Health (NIH) funded randomized controlled trials (RCT) [5]. Of the 142 included NIH-funded RCTs thirty-five studies limited enrollment to one sex. The median enrollment of women in the remaining 107 studies was 46%, but 16 enrolled less than 30% women. Only twenty-eight of the 107 (26%) studies reported at least one outcome by sex or explicitly included sex as a covariate in statistical analysis [5]. Surprisingly, even the Consolidated Standards of Reporting Trials (CONSORT) statement for randomized controlled trials does not include reporting results by sex [6].

Limited evidence is available on potentially relevant sex differences in peripheral and neuraxial regional anesthesia. Perhaps surprisingly, this deficiency is compounded by even less data on relevant sex differences in children, considering that boys are well known to require a number of common surgical procedures much more frequently. As a case in point, they outnumber girls by a factor of nine for inguinal hernia repair [7].

Even worse, potential sex differences in the incidence of critical events have not been addressed in large-scale prospective surveys on the safety of pediatric anesthesia [8]. Our group has recently furnished proof that caudal epidural blockade under sedation without airway instrumentation is a viable concept for standard use in the anesthetic management of subumbilical pediatric surgery [9]. As we did not focus on potential sex differences in that investigation, we designed a separate study for this purpose.

Hence, given the general scarcity of sex-specific data outlined above, we set out to identify in a retrospective observational study any sex differences in complication rates based on a population where, again, caudal blockade under sedation (without airway instrumentation) was the standard approach to subumbilical pediatric surgery.

The goal was to compare the sex-specific rates of success or failure for this primary anesthesia plan, of block-related technical complications, and of critical respiratory events. Any findings of a significant difference were to be evaluated against potential confounders to see if sex was independently associated with the variable in question.

## Materials and methods

The study was approved by the institutional review board (Ethics Commission at Medical University of Vienna; ref. 2370/2020; approved on 19-01-2021 and, upon amendment, on 21-07-2022) and entered in the German Clinical Trials Register (DRKS00024159; date of approval: 27-01-2021). Collected data were retrospectively analyzed and validated as per the STROBE guidelines [10]. The study was conducted at the Medical University of Vienna (Department of Anesthesia, General Intensive Care Medicine and Pain Medicine), a major tertiary-care centre with a catchment area of 3.5 million inhabitants. Considered for inclusion were all children (age: ≤ 15 years) admitted for pediatric surgery between 04/2014 and 12/2020, provided that caudal blockade under sedation only (i.e. without airway instrumentation) was the primary anesthesia plan. All procedures were done with written informend consent of a parent or legal guardian. As to the retrospective character of data extraction for this study, the need for consent of the parents or legal guardians for data extraction was waived by the ethics committee.

For premedication, we used midazolam (Dormicum™; Roche, Vienna, Austria) rectally at 0.5 mg kg^−1^ in 6-to-12-month-olds, orally at 0.5 mg kg^−1^ as a flavored syrup, or, if an intravenous access was already in place, via this route at 0.1 mg kg^−1^. The maximum allowed dosage was 15 mg midazolam orally or rectally and 5 mg for the intravenous route. Premedication was done at the discretion of the anesthesiologist. Infants less than six months of age were not premedicated. In cases without an intravenous line (predominantly patients aged ≤ 1 y), we induced mild sedation with inhalational sevoflurane up to 8 vol% via facemask to establish the intravenous line and stopped the sevoflurane immediately after placement. In older and compliant children, the intravenous line was placed without inhalational sedation. Cases with preexisting intravenous access and those after intravenous placement were sedated using propofol boluses at 1–2 mg kg^–1^ to facilitate caudal injection, if necessary, and continued intraoperatively at an infusion rate (not exceeding 5 mg kg^−1^ h^−1^) allowing the patient to spontaneously breathe and be aroused by significant physical stimulation. No use of continuous propofol was made for short (< 30 min) surgical or diagnostic procedures in up to 12-month-olds [11]. The sedated patients were supplied with oxygen-enriched air through a face mask attached by adhesive tape. Spontaneous breathing was continuously verified by an end-tidal CO2 line fitted to the face mask, through which oxygen-enriched air (FiO2: 0.40) was administered.

Patients were turned to left lateral with the hips and knees flexed to place the caudal block.

We take an approach to puncturing the sacral hiatus that combines anatomical landmarks with ultrasound guidance, preceded by establishing sterile conditions, including for the ultrasound probe (SaferSonic™; DanubiaMed, Persenbeug, Austria). For the caudal puncture, we used an ‘immobile needle technique’ with a needle featuring a short-bevel facet (30 mm, 24 G) and a prefilled 30 cm injection line (Pajunk, Geisingen, Germany). For real-time ultrasound visualisation of local anesthetic spreading into the epidural space, we place the sterile covered probe longitudinally in a position slightly paramedian to the lumbar spine cranial to the puncture site. This requires a three-hand technique: one anesthetist performs the puncture/injection (our standard caudal dosage being 1.0 ml kg^−1^ of ropivacaine 3.8 mg ml^−1^) and another one the visualisation.

We abandoned the primary anesthesia plan of neuraxial caudal blockade under sedation when we noticed a pain-related block failure at skin incision, defined by patient movement or a > 15% increase in heart rate from baseline, or any other event that called for general anesthesia with airway management. In these cases, a defined departmental standard of either endotracheal intubation or placement of a supraglottic airway device was instituted. The former consisted in careful bag-mask ventilation with < 10 mmHg of inspiratory pressure, followed by induction using propofol 2−4 mg kg^−1^, fentanyl 3−5 μg kg^−1^ and rocuronium 0.3–0.6 mg kg^−1^. The latter included propofol 2–3 mg kg^−1^ and fentanyl 2−3 μg kg^−1^.

Standard monitoring of each patient included electrocardiography, non-invasive arterial pressure, and peripheral oxygen saturation (SpO_2_) and an end-tidal CO2 line fitted to the face mask attached by adhesive tape.

Intravenous hydration with electrolytes was provided during the first hour of the surgical procedures at 10 ml kg^−1^ h^−1^ Elo-Mel isotone or, in under-1-year-olds, Elo-Paed balanced (Fresenius Kabi, Graz, Austria). After the first hour of the procedure the standard infusion rate of 10 ml kg^−1^ h^−1^ was adapted to 4 ml/kg/h for the 1–10 kg + 2 ml/kg/h for 11–20 kg + 1 ml/kg/h for > 20 kg bodyweight. Fluid boli were administered according to the specific demands of the individual patient.

All relevant data was retrieved from two independent electronic systems by systematic interrogation. The first of these was the electronic medical record (EMR) system used in all operating theatres and intermediate/intensive care units of our institution. The second system, whose name AKIM literally translates as General Hospital Information Management, is connected to a research, documentation and analysis database.

Both EMR and AKIM are separate patient documentation and information systems that operate in near real time and are quality assured by periodic data export and validation. The EMR system holds a copy of each anesthesia protocol since 2014 and collects a variety of data (e.g. on vital signs, medications, regional anesthesia, or airway devices). It features sections named *Primary and Secondary Anesthesia Plan* and *Unexpected (Critical) Events* for entry of complications like cardiac arrest, high spinal, laryngospasm, or bronchospasm. It is important to understand that attending anesthetists need to complete all required fields from within the operating theatre to close a case for ‘electronic transfer’ of the patient to the recovery room or intermediate/intensive care unit.

Three consultant anesthetists shared tasks in validating each of the clinical cases that were returned by the initial database search. Areas that were scrutinised for validation included patient characteristics, primary and secondary anesthesia plan, documented technique of regional anesthesia, use of an airway device and (if applicable) ventilation parameters, sequence of steps and events in the operating theatre, critical events, lowest heart rate, lowest SpO_2_ value, discharge records, anesthesia consent form, as well as (if applicable and needed) digital records from the intermediate/intensive care units.

Meticulous attention was devoted to identifying artefacts, verifying the factuality of complication(s), establishing whether the primary anesthesia plan had been followed through, and filling in any blanks from different sources (e.g. whenever values for body weight were missing). This first round for each case resulted in a rating of A to E:

A The primary plan was caudal epidural anesthesia without airway instrumentation and clearly was followed through in a straightforward and uneventful process

B The primary plan was caudal epidural anesthesia without airway instrumentation but clearly was abandoned for general anesthesia

C An event was noted (e.g. cardiac arrest, high spinal, laryngospasm, bronchospasm)

D The primary plan clearly was not caudal epidural anesthesia; rather, the epidural was provided as an adjunct to a primary plan of general anesthesia

E Case needs reassessment

**Fig 1:**
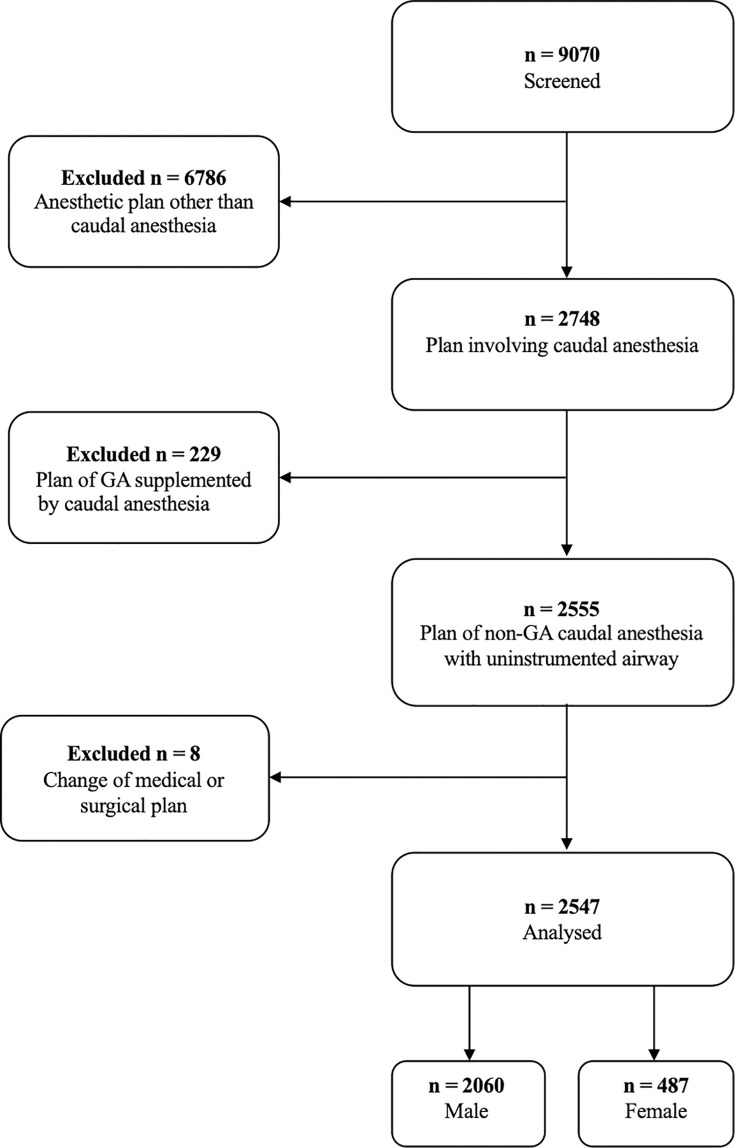
Flow chart illustrating the roadmap of database interrogation for this study (GA general anesthesia).

After this first round of validation, cases in category D were excluded (see Fig 1). The purpose of the second round was to have all cases in categories B, C and E reassessed by a different examiner than in the first round, followed by a last round in which all cases in categories C and E were referred to the most experienced anesthetist for final assessment. It was also up for this clinician to settle any disagreement on whether a case had been converted to general anesthesia due to pain, respiratory failure, or other events.

As primary outcome parameter, we defined the sex-specific failure rates involved in the primary anesthesia plan (caudal blockade under sedation with an uninstrumented airway). This endpoint was reached when the primary plan was abandoned for the contingency plan of general anesthesia at skin incision. Secondary outcomes included the sex-specific rates of residual pain, of block-related technical complications (blood aspiration, high spinal, anatomical impediments (defined as impossibility to perform the caudal block due to any kind of obstruction of the hiatus sacralis (e.g. due to bony or bony-like structures), subcutaneous injection of local anesthetic), and of critical respiratory events (laryngospasm, bronchospasm, apnoea, cardiac arrest to hypoxia, or any less well specified events requiring action by the anesthetist).

To arrive at valid comparisons between the rates at which these primary and secondary outcomes occurred in boys versus girls, significant findings of sex differences in these rates were followed by using the same combined dichotomous (yes/no) endpoint (defined as at least one event per case) in a logistic regression analysis against potential confounders disclosing if sex was independently associated with this category of events.

Logistic regression results are reported as raw and confounder-adjusted odds ratios with 95% confidence intervals. Specific variables were defined as potential confounders on the basis of established relationships between explanatory and outcome variables, biological plausibility, and inhomogeneous distribution at p < 0.1. Once a co-variable was included in the multiple regression model, a stepwise logistic regression was performed to assess any association between sex and the combined dichotomous (yes/no) endpoint for its standalone quality. Prior to including co-variables, they were tested for interactions and collinearity, including the use of variance inflation factors (1/1-R_i_^2^) [12].

All data were pre-screened for completeness, consistency, and outliers. Where values were missing, we explored alternative sources of data before considering imputation. If ≤ 5% of values were then still missing, we replaced them with appropriate subgroup medians. Continuous data were evaluated using a non-parametric Mann-Whitney U-test. Categorical variables are presented as counts and cross-tab calculations compared with Fisher’s or Pearson’s chi-square test. The 95% binomial confidence interval (CI) for the incidence of complications was calculated using the Jeffreys method. All tests were carried out two-sided, considering differences to be significant at p < 0.05 and adjusting multiple hypotheses for α-error accumulation by Bonferroni correction. The software environments used for all operations in our analysis were SPSS® Statistics (version 24.0.0.0, IBM, Armonk, NY, USA) and R (R-Foundation, Vienna, Austria).

## Results

Fig 1 depicts a flow chart of the inclusion and exclusion process for this study. Of 9070 screened cases, a total of 2547 could ultimately be evaluated, 2060 being boys and 487 being girls.

As apparent from the demographic and anesthesia-related data summarised in Table 1, the girls were significantly older (p < 0.025) while the boys included a significantly higher proportion of preterm births (p = 0.009).

**Table 1:** Sex differences in demographic and anesthesia-related patient characteristics.

	Boys (N = 2060)		Girls (N = 487)		p-value
Chronological age (weeks)	76.6	(28–232)	125.4	(24–257)	< 0.025^a^
ASA physical status					0.1^b^
1	1567	(76.1%)	356	(73.1%)	
2	347	(16.8%)	99	(20.3%)	
3	144	(7%)	30	(6.2%)	
4	2	(0.1%)	2	(0.4%)	
Body weight (kg)	11	(7.2–17.3)	12.5	(6.8–18)	0.41^a^
Body weight ranges (kg)					< 0.001^b^
0 to < 5	263	(12.8%)	84	(17.2%)	
5 to < 10	573	(27.8%)	102	(20.9%)	
10 to < 20	842	(40.9%)	208	(42.7%)	
20 to < 30	295	(14.3%)	86	(17.7%)	
30 to < 40	87	(4.2%)	7	(1.4%)	
Bronchopulmonary dysplasia (yes/no)	32	(1.6%)	3	(0.6%)	0.13^c^
Gestational week at birth					0.009^b^
< 28 (extreme preterm)	126	(6.1%)	16	(3.3%)	
28 to < 37 (very-late preterm)	262	(12.7%)	49	(10.1%)	
> 37 (term)	1672	(81.2%)	422	(86.7%)	
Previous caudal blocks					0.97^b^
None	1819	(88.3%)	431	(88.5%)	
One caudal block	179	(8.7%)	41	(8.4%)	
≥ 2 caudal blocks	62	(3%)	15	(3.1%)	
Attempts to successful caudal block (n)	1	(1–2)	1	(1–2)	0.98
Amount of ropivacaine 0.375% (ml)	11	(7.5–17)	13	(7–19)	0.29^a^
Caudal block to skin incision (min)	12	(9–16)	12	(9–16)	0.53^a^
Surgical procedure time (min)	32	(20–50)	22	(15–31)	<0.0001

Data median values with interquartile ranges (IQR) or absolute numbers with percentages (%).^a^Mann-Whitney U-test; ^b^Peason’s chi-square test; ^c^Fisher´s exact test; CB = caudal blockade.

The failure rate of caudal blockade without airway instrumentation was 5.5% (95%CI 3.8%-7.8%) among the girls (*N* = 27 of 487) and 4.7% (95%CI 3.9%-5.7%) among the boys (*N* = 97 of 2060), which was not found to be a significant difference between the sexes (p = 0.41).

Residual pain was the most common reason for switching over to the contingency plan of general anesthesia. This came to pass in 22 of the girls (4.5%, 95%CI 2.9–6.6%) and 61 of the boys (3.0%, 95%CI 2.3–3.8%), likewise not involving a significant difference (p = 0.089).

6 (0.24%,(95%CI 0.1–0.5%)) cases of anatomical impediments did occur with the result that caudal blockade could not be performed and our primary anesthesia plan was abandoned for general anesthesia immediately prior to skin incision. All of these cases were male patients.

**Fig 2:**
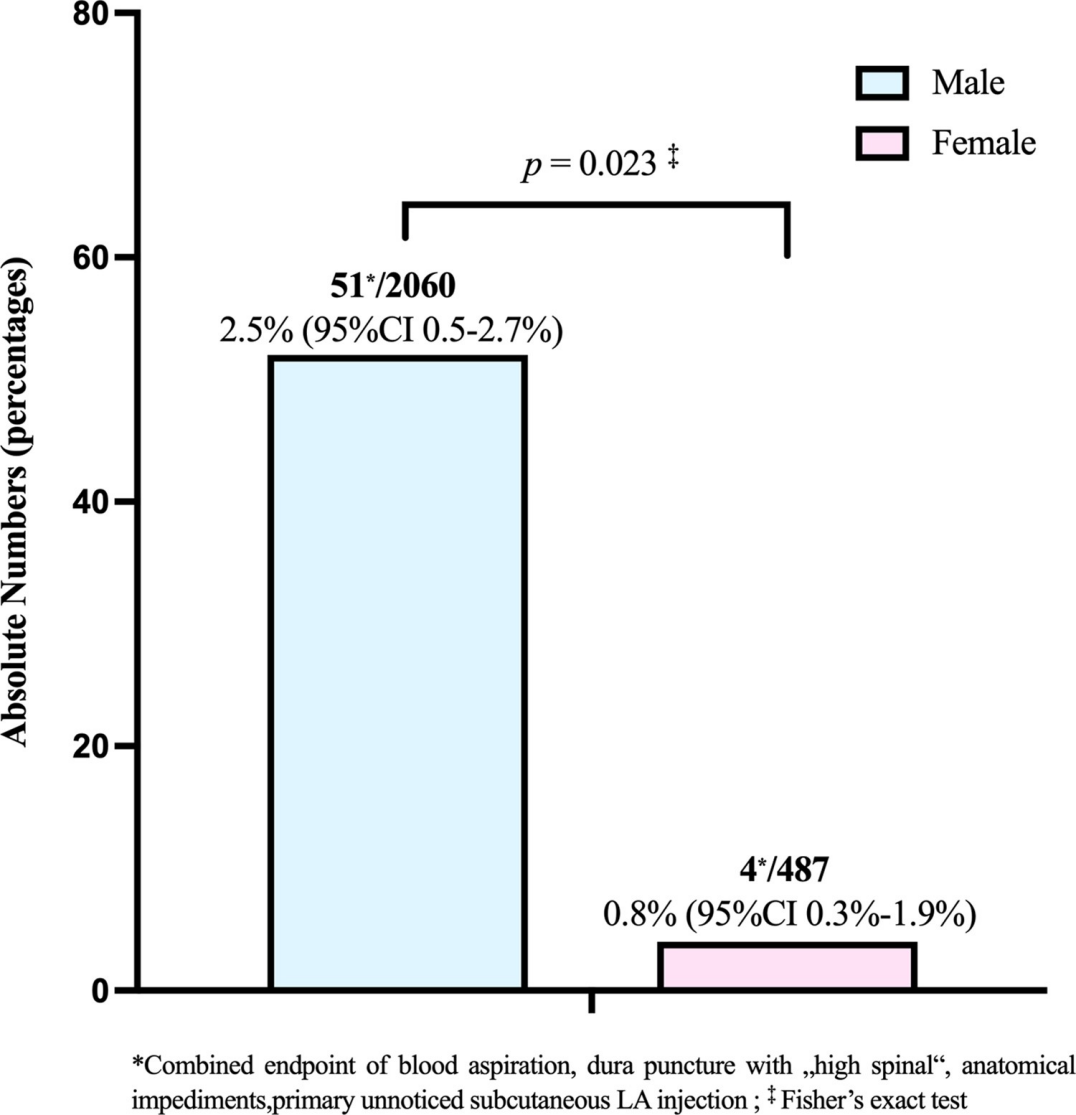
Bar graph of any block-related technical complications in boys versus girls. *Combined endpoint of blood aspiration, dura puncture with „high spinal“, anatomical impediments, primary unnoticed subcutaneous LA injection; ^‡^ Fisher’s exact test. Given are absolute numbers and percentages including 95% binomial confidence interval (CI) for the total sex-specific rates.

As shown in Fig 2, the combined endpoint of block-related technical complications applied to 55 patients overall, with rates of 2.5% (95%CI 0.5–2.7%) among the boys (*N* = 51) versus 0.8% (95%CI 0.3%-1.9%) (*N* = 4) among the girls indicating a significant sex difference (p = 0.023).

**Fig 3:**
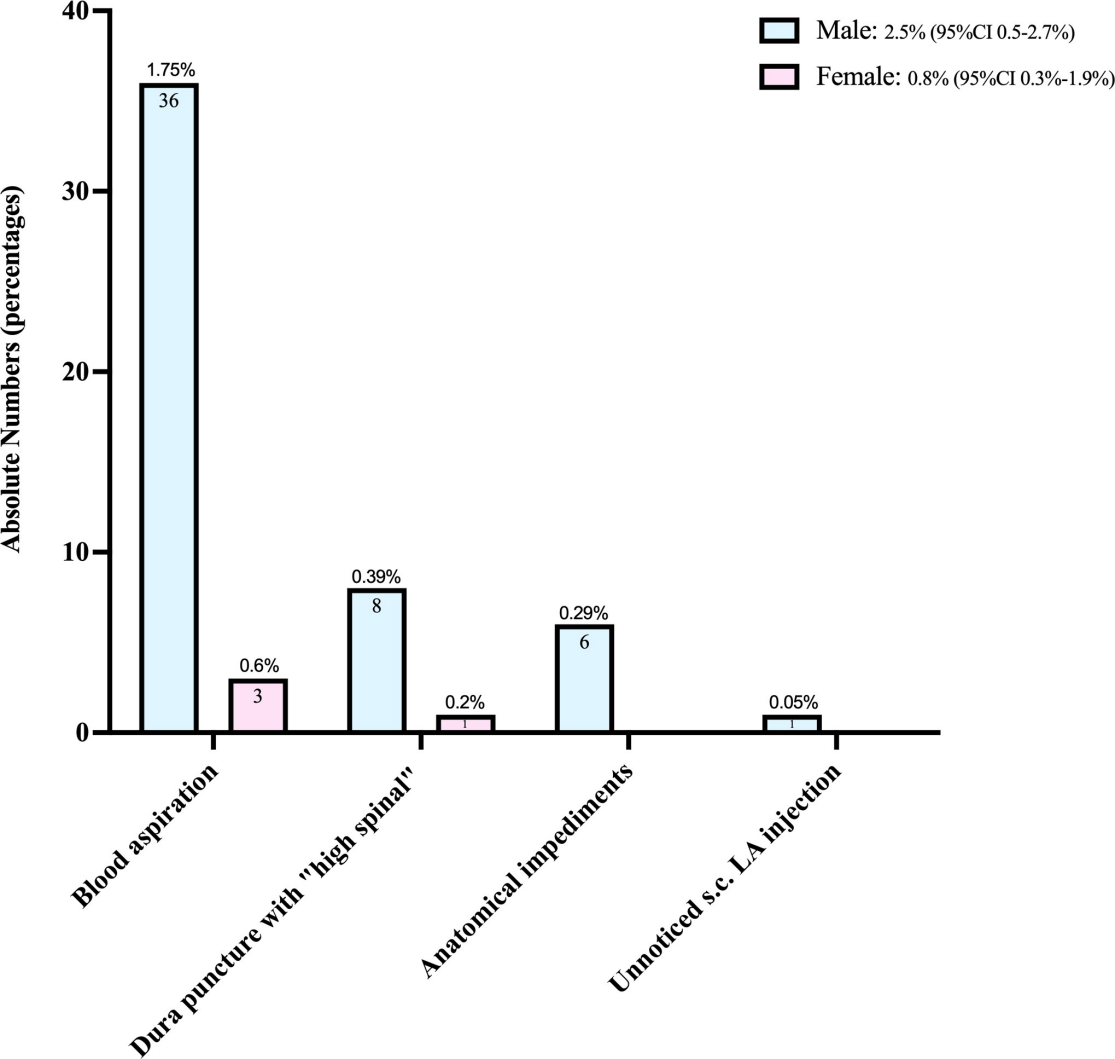
Specific block-related technical complications in 2060 boys and 487 girls. Given are absolute numbers and percentages including 95% binomial confidence interval (CI) for the total sex-specific rates.

Fig 3 breaks down in absolute terms the distribution of specific event types that were pooled within this combined endpoint and illustrates the sex differentials involved.

Based on our multivaribale logistic regression model, which is summarised in Table 2, male sex was significantly associated with higher odds (adjusted Odds Ratio: 3.18; 95% confidence interval: 1.12−9; *p* = 0.029) for this combined endpoint of block-related technical complications. Note that this finding was obtained regardless of chronological age, ASA physical status, or gestational week at birth. The numbers of puncture attempts (n) to a successful caudal blockade was significant associated with higher odds for the combined endpoint of block-related technical complications and served as an effect modifier in regard to the association of sex with outcome (Table 2). Body weight, due to its collinearity with chronological age, was excluded from this analysis (Table 2).

**Table 2:** Logistic regression results for sex-specific associations with block-related technical complications^a^.

	Univariable analysis (crude)			Multivariable analysis (adjusted)		
	Odds ratio (95% CI)		p- value	Odds ratio (95% CI)		p-value
Male sex	3.065	1.1–8.5	0.032	3.18	1.12–9	0.029
ASA physical status ≥ 2 *vs* 1	0.76	0.42–1.38	0.55	0.7	0.38–1.35	0.3
Weight (kg)	1	0.97–1.04	0.83	n/a (collinearity)^b^		VIF = 7.28
Chronological age (weeks)	1.0	0.99–1	0.86	1.0	0.99–1.002	0.98
Attempts to successful caudal block (n)	2.65	2.1–3.4	<0.001	2.7	2.1–3.5	<0.001
Prematurity compared to full term						
< 28 weeks of gestation	1.78	0.69–4.57	0.23	1.78	0.64–4.9	0.26
28 to < 37 weeks of gestation	1.29	0.6–2.77	0.51	1.49	0.66–3.36	0.33

^a^Cases of blood aspiration, dura puncture with high spinal, anatomical impediments, or subcutaneous injection of local anesthetic; ^b^collinear variables are body weight and chronological age; VIF = variance inflation factor.

**Fig 4:**
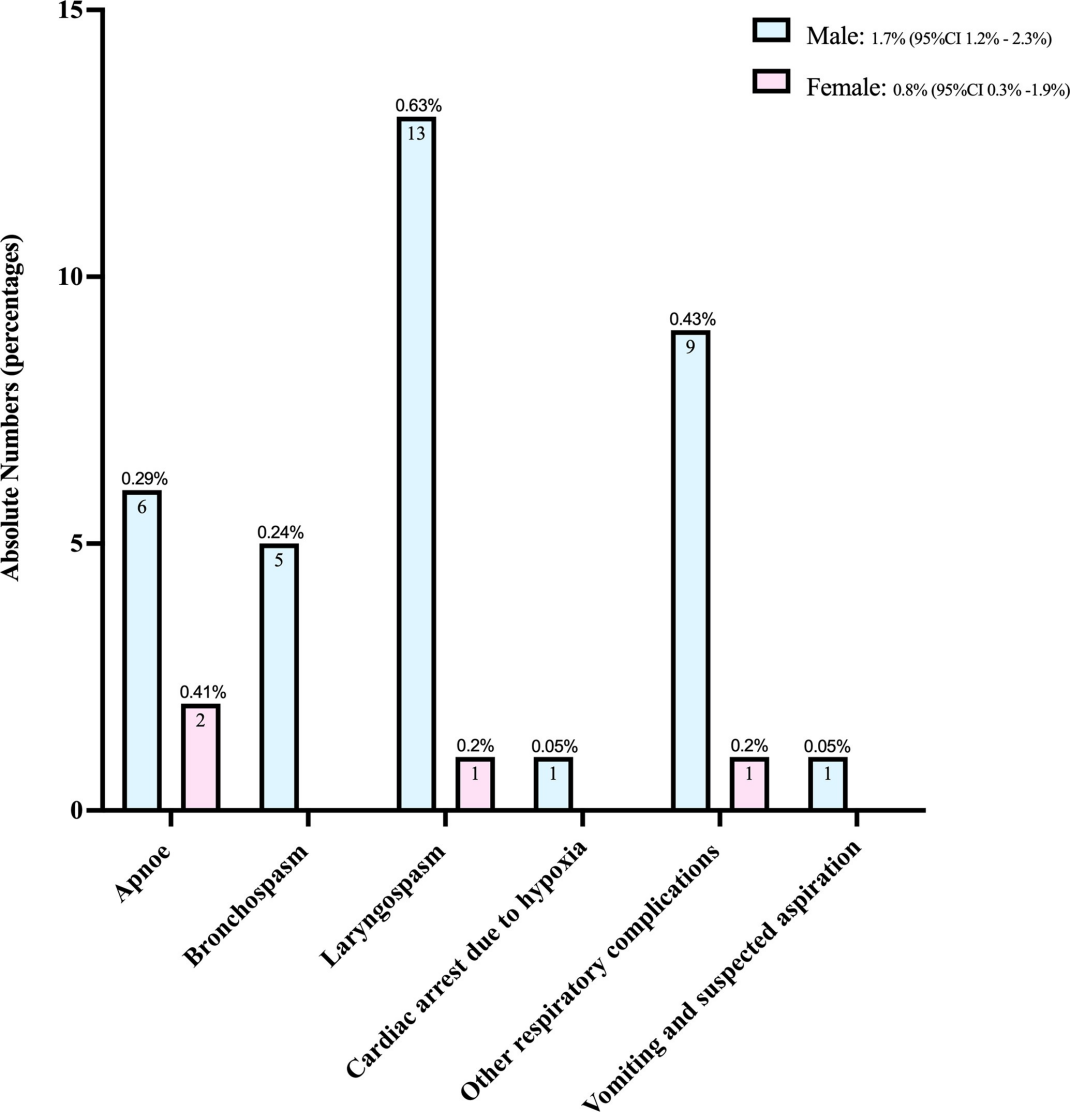
Bar graph of any critical respiratory events in 2060 boys and 487 girls. Given are absolute numbers and percentages including 95% binomial confidence interval (CI) for the total sex-specific rates.

While the rate of critical respiratory events was, at 1.7% (95%CI 1.2%-2.3%) (*N* = 35), more than twice as high among the boys than, at 0.8% (95%CI 0.3%-1.9%) (*N* = 4), among the girls, the numbers involved did not amount to a statistically significant difference between the sexes (*p =* 0.21). Fig 4 breaks down the distribution of specific events within this category in absolute terms and illustrates the sex differentials involved.

## Discussion

In this first study to explore sex differences involved in the most commonly used technique of pediatric regional anesthesia, we found that male sex was significantly associated with higher odds for caudal block technique related complications by a factor of three in comparison to girls. A noticeably, albeit not significantly, higher rate was also observed for critical respiratory events among boys.

Another finding underscoring the greater challenges of caudal anesthesia in male patients, regardless of the chronological age brackets in our logistic regression model, is that all six block attempts that failed for anatomical reasons did so in boys. As an aside, this very low anatomy-related failure rate of 0.24% (95%CI 0.1–0.5%) (given 2547 cases overall) bears testimony to our experience that visualisation by ultrasound can effectively minimise the risk of misplacing punctures or of unexpectedly meeting with anatomical impediments. Ultrasound imaging is an increasingly used tool of advanced pediatric anesthesia and is extremely useful for caudal blocks in small children [9, 13]. In the present series, all 2547 evaluable cases (100%) were successfully covered by ultrasound visualisation. According to a recent RCT by Ahiskalioglu and coworkers the use of ultrasound reduces the complications such as vascular puncture or subcutaneous tissue bulging and increases the success rate of first puncture in pediatric caudal injection compared to conventional technique without ultrasound use. These authors report in the setting of RCT a block success rate of 97% with ultrasound and 93% without [14]. A recent systematic review underlines this finding. So, ultrasound-guided caudal injection results in higher first puncture success and lower incidence of complications compared to landmark technique [15]. However, data of a RCT are with difficulty compareable to our data being a reflection of caudal blocks in children of different ages and routinely performed by anesthetists with various levels of training and not–as in RCT–by experts. Our data may be encumbered by accuracy and quality concerns appertaining to real-time documentation and different levels of experience, but we believe that the value of real-life routine management being more truly reflected may outweigh these concerns [9].

The lower rates for caudal block associated complications seen in girls might hypothetically, at least in part, be explainable to sex differences in body composition and anatomy. Compared to biological males, females exhibit a greater range of lumbar spine mobility during ontogenetic development (investigated age range: 3−25 years) [16]. Similarly, newborn girls have been found to exhibit greater flexibility of the spine due to, on average, significantly smaller cross-sectional dimensions of vertebrae compared to boys [17]. This greater flexibility might help us to maximise flexing of the neck, hips and knees for lateral positioning of female patients during caudal blockade. Also, it might be easier in girls to spot landmarks for caudal blockade, given the result—emerging from an ultrasound imaging study of foetal ossification during the first and second trimesters of pregnancy—of a significantly earlier start of ossification in females than males [18].

Findings of a significantly increased thickness of the sacrococcygeal ligament in male compared to female patients, even though that study was based on adults [19], might render puncturing of the sacral hiatus more difficult in boys than girls. In addition, the higher incidence of high spinals among the boys in our series might be explainable by a shorter distance between the upper margin of the sacrococcygeal membrane and the dural sac in boys, as reported in a pediatric magnetic resonance image study [20].

Compared to girls, we also observed a higher proportion of blood aspirations among the boys during caudal blockade, and one might speculate that the risk of vessel puncture may be a function of sex differences in the morphology of the epidural venous plexus and in the distribution or proportion of epidural fat. Even though data on sex differences in epidural space composition is currently not available, studies in full-term neonates show that differences in body composition are, in fact, present at birth, with female sex being a strong predictor of body fat percentage [21].

Whether sex differences may exist in the lumbosacral anatomy of the internal vertebral venous plexus (IVVP) is unclear. Groen et al. [22] reported age-related morphological features of the IVVP, without identifying a prominent posterior lumbar IVVP in fetuses 21–25 weeks of gestational age comparable to the one in adults. They assumed that its caudal portion changes significantly during the remaining months of intrauterine life and that the absence of a prominent posterior lumbar IVVP may protect newborns from heat loss. Possibly, therefore, the risk of IVVP puncture and blood aspiration involved in caudal epidurals may be a function of age-related IVVP development, whereas the authors of that study did not comment on sex differences in lumbar IVVP morphology [22].

Some of the girls in our study might have benefitted from higher doses of ropivacaine, given that pain (patient movement or > 15% increase in heart rate) both was the most common reason for switching over to general anesthesia overall and involved a higher incidence among the girls than the boys (p = 0.089). Also, female patients have been found to require a minimum concentration of ropivacaine for caudal anesthesia 31% higher than male patients, amounting to 0.389% (95% CI; 0.372%–0.407%) [23]. The 0.375% concentration in our series, with comparable total amounts of ropivacaine administered to both sexes, appears to support this notion that a minority of girls would benefit from even higher concentrations. Furthermore, a report on spinal anesthesia in infants, while demonstrating no statistically significant evidence of sex differences for levobupivacaine and ropivacaine at the ED50 and ED95 dose ranges, did find a ED50 for ropivacaine 0.5% twice as high for girls (0.64 mg.kg^−1^) as for boys (0.3 mg.kg^−1^) [24].

Compared to the girls in our series, and given a low overall incidence of 1.53% (95%CI 1.1–2.1%) (*N =* 39/2547), the rate of respiratory events was more than twofold among the boys (*p =* 0.21). This may be due to the higher proportion of extreme preterms among the boys, considering that a sex difference—specifically characterised as a “male disadvantage”—is well documented for neonatal outcomes after preterm births [25]. In addition, there is evidence for a sex difference in airway structure in infants, with a smaller airway size in boys resulting in a 20% higher maximum flow at functional residual capacity in girls during the first year of life [26]. Greater bulk of smooth muscle and thickness of the inner airway wall in boys has been suggested to account for some of these sex differences in airway function and susceptibility to respiratory disease in early life [26, 27]. Another factor plausibly contributing to the higher respiratory susceptibility of the boys in our series was their significantly younger age at the time of surgery (*p* < 0.025; see Table 1).

Our data should be interpreted cautiously. The presented data set represents the largest single centre experience of caudal blockade under sedation without primary airway manipulation. Fortunately, the rate for critical events was low. However, this limits the statistical power of our adjusted analyses. Furthermore, other unknown confounders or effect modifiers may have affected the adjusted analyses. Particularly, the experience of the performing anesthesist might be of interest in this context [28, 29]. However, we deem it unlikely, that rather unexperienced anesthetists performed proportionally more caudal blocks in male patients. However, we cannot support this assumption reliably with our dataset. Our stringent validation process should have left little space to overlook severe complications but naturally cannot eliminate the risk of non-reporting and detection bias or differences between anesthesia teams in judging clinical situations. Additionally, the presented data are a reflection of caudal blocks in children of various ages and routinely performed by anesthetists with different levels of training. Furthermore, as noted above, boys are well known to require a number of common surgical procedures much more frequently. This fact created a sex-imbalance towards male sex in our series, however this is a common issue in pediatric observational studies [30]. Nevertheless, this imbalance may be a source of selection bias. Moreover, the positive association of male sex with higher odds of block-related technical complications cannot demonstrade a causal relationship. As our departmental standard for subumbilical surgery is, indeed, caudal blockade under sedation with an uninstrumented airway, our data cannot be readily compared with previous cohorts, which predominantly reflect either combinations with general anesthesia [31].

## Conclusions

A matter as trivial as patient sex is really the beginning of personalized medicine, and this triviality has not always received the attention it deserves. The results of our study reaffirm that sex is an important consideration integral to any assessment of individual risk in pediatric patients. Its main finding that, compared to girls, boys are more likely to experience both block-related technical complications and critical respiratory events is consistent with data on pediatric sex differences unrelated to anesthesia. A recent study has hinted at potential sex differences in neurodevelopment after neonatal exposure to general anesthesia [2]. Hence techniques of regional anesthesia may be preferable in these situations, but establishing best practices in early-childhood anesthesia will require further research to develop a more comprehensive body of data on relevant anatomical, pharmacological, and physiological sex differences.
